# En Bloc Orbitectomy in the Management of Advanced Orbital Cancer: A Retrospective Study of Seven Different Carcinomas

**DOI:** 10.7759/cureus.79380

**Published:** 2025-02-20

**Authors:** Lukas Hauer, Peter Tvrdy, Walla Samara, Petr Posta, Zdenek Kasl, Inka Treskova, Jiri Gencur, Omid Moztarzadeh

**Affiliations:** 1 Department of Stomatology, University Hospital Pilsen, Faculty of Medicine in Pilsen, Charles University, Pilsen, CZE; 2 Department of Oral and Maxillofacial Surgery, University Hospital Olomouc, Olomouc, CZE; 3 Clinic of Ophthalmology, University Hospital Pilsen, Faculty of Medicine in Pilsen, Charles University, Pilsen, CZE; 4 Department of Plastic Surgery, University Hospital Pilsen, Faculty of Medicine in Pilsen, Charles University, Pilsen, CZE; 5 Department of Anatomy, Faculty of Medicine in Pilsen, Charles University, Pilsen, CZE

**Keywords:** carcinoma, en bloc orbitectomy, extended orbital exenteration, orbital exenteration, orbital invasion, orbital tumors

## Abstract

Aim: An en bloc orbitectomy is one of the most invasive surgical procedures in the orbit, primarily used to remove advanced malignancies, ensure negative resection margins, and local cancer control. This study examines the indications, clinical features, outcomes, and survival rates of patients who underwent this surgery for periocular and orbital malignancies.

Patients and methods: A retrospective consecutive case series of seven patients with various orbital carcinomas managed with en bloc orbitectomy from 2018-2023 at the University Hospital in Pilsen, Czech Republic, was conducted. Minor surgeries and other diagnoses were excluded.

Results: The study included seven patients, with an average age of 68.7 years (range 50-83, median 74). Orbitectomy was part of salvage surgery in two patients and debulking in one. R0 resection was achieved in only 50% of cases; defects were reconstructed with a free anterolateral thigh (ALT) flap in one case and local flaps in others. Therapeutic neck dissection was performed in three patients directly after surgery, in one case 15 months after orbitectomy.

Conclusion: A thorough examination of indications, patient and tumor features, and reconstructive possibilities should precede an en bloc orbitectomy. Despite being highly invasive, it should be adequately indicated to avoid less invasive surgeries that could complicate radical surgery and impair disease control.

## Introduction

A wide variety of benign and malignant tumors can arise in the orbit, which may occur as a primary tumor, secondary because of extension from nearby structures, or as a result of distant metastases [1]. Malignant tumors represent about 32-36% of all orbital space-occupying lesions and can arise from a variety of origins. Primary tumors of the lacrimal apparatus, such as adenoid cystic carcinoma; primary tumors of mesenchymal origin, like rhabdomyosarcoma; primary tumors of the optic nerve, such as malignant optic nerve glioma; and secondary tumors originating from nearby structures, such as the nasal cavity, paranasal sinuses, facial skin, and anterior cranial fossa, necessitate a broader surgical approach [2,3].

Primary orbital malignancies are uncommon and are generally characterized according to the originating tissue type. In adults, the most common primary malignant orbital tumors are lymphoproliferative lesions of the orbit and adnexa, constituting up to 20% of all orbital masses [1]. Identifying orbital diseases at earlier stages and correcting them with dedicated imaging enables the most effective therapy, whether therapeutic or surgical, to maintain vision and limit complications. For orbital imaging, ultrasound, computed tomography (CT), and magnetic resonance imaging (MRI) are commonly used. High-resolution MRI of the orbit has recently led to the diagnosis of an increasing number of orbital diseases and tumors [4]. To successfully treat extensive tumors, physicians with expertise in head and neck surgery, neurosurgery, ophthalmology, plastic surgery, radiation, and medical oncology need to collaborate [3]. Radiation, surgery, chemotherapy, hormonal therapy, targeted therapy, and immunotherapy, or a combination of these methods, may be used as a form of treatment [5]. In most cases, surgery is still a valuable diagnostic and/or therapeutic choice for orbital tumors. Before more conclusive treatment is applied, a biopsy is frequently carried out for histopathologic confirmation of suspected malignant lesions. The tumor's type, size, location, closeness to important tissues, and the intended outcome of the surgery all play a major role in the surgical technique selection [1].

In cases such as orbital lymphoma, where symptoms can be nonspecific, an early surgical biopsy is essential for prompt diagnosis. After confirming the diagnosis and completing staging, radiation and/or systemic therapy are typically more effective as initial treatments. Therefore, orbital surgery is generally not recommended, as it may lead to significant visual complications and is not considered a therapeutic approach. However, in some situations, including but not limited to these malignant tumors, partial tumor resection may be necessary to alleviate patient discomfort and help restore visual function [6,7].

Orbital exenteration is an extensive surgical procedure that involves removing the eye, extraocular muscles, and surrounding soft tissue in the orbit. In rare cases, the procedure may also involve the removal of some or all of the orbit's bony walls, a procedure known as extended orbital exenteration or en bloc orbitectomy, either with or without the preservation of the eyelids. These procedures, which can be both psychologically and anatomically disfiguring, are typically reserved for certain primary orbital malignancies or tumors that have spread from adjacent areas, particularly for carcinomas [8,9]. There is no controversy regarding the need for orbital exenteration in cases of significant orbital tumor invasion or when the extraconal muscles and orbital apex are involved [10]. An en bloc orbitectomy is indicated when the tumor is attached to or invades the bones of the orbit [11], even in patients with minimal bone or periosteum infiltration, to obtain clear margins [10]. In a broad sense, an orbitectomy involves the removal of any part of the orbital wall, along with the complete removal of orbital contents (en bloc orbitectomy or extended orbital exenteration), partial removal (typically eye-sparing orbitectomy), or without removing any contents. This procedure is performed to treat certain diseases that have spread from the orbit to surrounding areas, or conversely, from surrounding areas into the orbit. Orbitectomy is a disruptive treatment that may involve surgery in the nasal sinuses, jaw, skull, and cranial cavities [12].

In contrast to this extensive surgical treatment, new paradigms for eye-sparing cancer treatments are starting to take shape. These can be based on conservative surgery with or without additional radiotherapy, targeted therapies, or immunotherapies. This approach prioritizes maintaining vision and aesthetic appearance while effectively managing the disease. It represents a shift towards more conservative and targeted interventions, improving patient outcomes and quality of life [9].

Therefore, the objective of this study is to assess a group of patients who received complex therapy for seven distinct kinds of primary or secondary orbital carcinoma, which required en bloc orbitectomy. The purpose is to compare the clinical outcomes and prognosis of each patient following the procedure.

## Materials and methods

This retrospective study was conducted to evaluate the clinical outcomes of en bloc orbitectomy procedures performed for various primary and secondary orbital carcinomas. A total of seven patients who underwent this surgery were included in the analysis. The study was carried out at the Stomatology Department of the University Hospital in Pilsen, Czech Republic, over a period of nearly six years, between February 2018 and December 2023. The mean follow-up duration was calculated to be 27.9 ± 22.8 months, with follow-up periods ranging from 0 to 67 months, allowing for comprehensive post-operative evaluation.

The primary aim of the study was to assess key surgical outcomes, including recurrence rates, overall survival rates, and the incidence of post-operative complications. Additional objectives included evaluating tumor characteristics, the extent of surgical resection achieved, and the role of adjuvant therapies. These data were systematically analyzed to identify factors that may influence clinical outcomes and patient prognosis following en bloc orbitectomy.

Inclusion criteria

Patients selected for inclusion in the study were strictly limited to those diagnosed with orbital carcinoma who were treated with en bloc orbitectomy. Cases involving less extensive surgical procedures, minor interventions, or alternative differential diagnoses were excluded. This ensured that the findings specifically reflected the outcomes of patients undergoing this surgical technique for orbital carcinoma.

Data collection and review

The clinical data collected for each patient included detailed records of tumor characteristics, histopathological findings, and imaging studies. Tumor staging and diagnoses were confirmed using advanced imaging modalities, such as CT, F-18 fluorodeoxyglucose (FDG) positron emission tomography (PET)/CT, 18F-FDG PET/MR and histological examination of surgical specimens. Additionally, the study reviewed radiographic images, treatment modalities employed, the presence of metastatic disease, and the extent of tumor resection. These records were carefully analyzed to ensure accuracy and reliability in the evaluation of clinical outcomes.

Tumor characteristics and diagnoses

The diagnosis of orbital carcinoma was categorized based on histopathological findings, revealing a diverse spectrum of primary and secondary orbital malignancies. The carcinomas identified included the following: moderately to poorly differentiated unspecified carcinoma (primary orbital carcinoma); adnexal apocrine adenocarcinoma (secondary orbital carcinoma); sebaceous carcinoma (secondary orbital carcinoma); squamous cell carcinoma (secondary orbital carcinoma); mucoepidermoid carcinoma (secondary orbital carcinoma); clear cell myoepithelial carcinoma (primary orbital carcinoma); and basal cell carcinoma (secondary orbital carcinoma).

Study goals

This research focuses on evaluating the success of en bloc orbitectomy in treating orbital carcinomas by examining outcomes such as surgery results, recurrence rates, survival statistics, and complications after the procedure. The study's findings aim to improve surgical methods and contribute to creating customized treatment plans for these complex conditions.

The Ethical Committee of the University Hospital Pilsen and the Faculty of Medicine in Pilsen, Charles University, approved the study protocol with reference number 248/24.

## Results

This retrospective study assessed seven patients with an average age of 68.7±10.8 years (range 50-83 years, median 74 years). The ratio of men to women was 2:5.

Each patient underwent an en bloc orbitectomy with different extensions (lateral, medial, cranial, and caudal). Three patients underwent medial and caudal extensions, while one patient underwent lateral, medial, and caudal extensions.

Two patients underwent salvage surgery, one with cranial extension and the other with cranial, caudal, and medial extension. A patient of advanced age with a multi-year history and slow progression of low-grade myoepithelial carcinoma in the left orbit, resulting in the displacement of the eyeball and recurrent infections, underwent a debulking procedure. This surgery involved the cranial and lateral walls, extending only to the frontal sinus. Given the tumor's type, its slow progression, and the patient's age, a radical surgery involving en bloc orbitectomy and resection of the skull base with intracranial involvement was not indicated.

R0 resection was achieved in only 50% of the patients, in whom the intention was radical tumor removal, and R1 resection was achieved in the rest of the cases. Two patients who had imaging evidence of metastasis at the time of diagnosis underwent a therapeutic en bloc dissection of neck lymph nodes in the range of ND R/L (I-V) immediately after orbitectomy, and one patient underwent this procedure 15 months after orbitectomy when 18F-FDG PET/CT during follow-up revealed suspicion of metastases.

In one case, the defect was reconstructed with a free anterolateral thigh (ALT) flap. In other cases, the repair was done using a temporalis muscle flap, either in combination with local skin flaps or using only the latter. An orbital epithesis was used in one patient.

After the procedure, three patients received radiotherapy: two as adjuvant therapy and one as treatment for recurrence.

The average follow-up was 27.9 months (range: 0-67 months). From an oncological point of view, the results were as follows: three cases with no evidence of disease (NED), two cases died of other causes (DOC), one case died of disease (DOD), and one case was alive with a local recurrence of the disease (AWD).

Table 1 summarizes the clinical outcome and results for each patient after the surgical procedure.

**Table 1:** Comparison of each patient's clinical findings and prognosis after surgery Abbreviations NED (No evidence of disease), DOD (Died of the disease), DOC (Died of other cause), M1 – OSS (Distant Metastasis  – Osseous), M1 - BRA (Distant Metastasis  – Brain), AWD (Alive with disease), R0 (Microscopically negative margins), R1(Microscopically positive margins), ALT (Anterolateral thigh), RT (Radiotherapy), S (Salvage surgery), D (Debulking surgery).

Patients	Sex	Age	Diagnosis	En bloc orbitectomy -bony wall	Resection	Reconstruction	Neck dissection	Oncology therapy	Follow-up (months)	Results
1	F	76	Moderately to poorly differentiated unspecified carcinoma	Medial and caudal	R0	Temporalis muscle flap+ local skin flaps + epithesis	After 15 months; ND R(I-V)	2x Adjuvant RT	67	NED
2	M	59	Adnexal apocrine adenocarcinoma	S cranial	R1	Temporalis muscle flap +local skin flaps	ND L (I-V)	-	24	DOD (M1-OSS, BRA)
3	F	63	Sebaceous carcinoma	Medial and caudal	R1	Local skin flaps (glabellar flap + cheek rotation advancement flap)	-	Adjuvant RT	5	DOC (meningitis)
4	F	76	Squamous cell carcinoma	Lateral, caudal and medial	R0	Temporalis muscle flap+ cervicofacial flap	ND R (I-V)	-	0	DOC (sepsis)
5	M	50	High-grade mucoepidermoid carcinoma	S cranial, caudal and medial	R1	ALT flap	-	After 24 months, reRT (recurrence)	39	NED
6	F	83	low-grade myoepithelial carcinoma	D cranial and lateral	R1	Temporalis muscle flap+ local skin flaps	-	-	48	AWD (local recurrence)
7	F	74	Basal cell carcinoma	Medial and caudal	R0	Local skin flaps	-	-	12	NED

**Figure 1:**
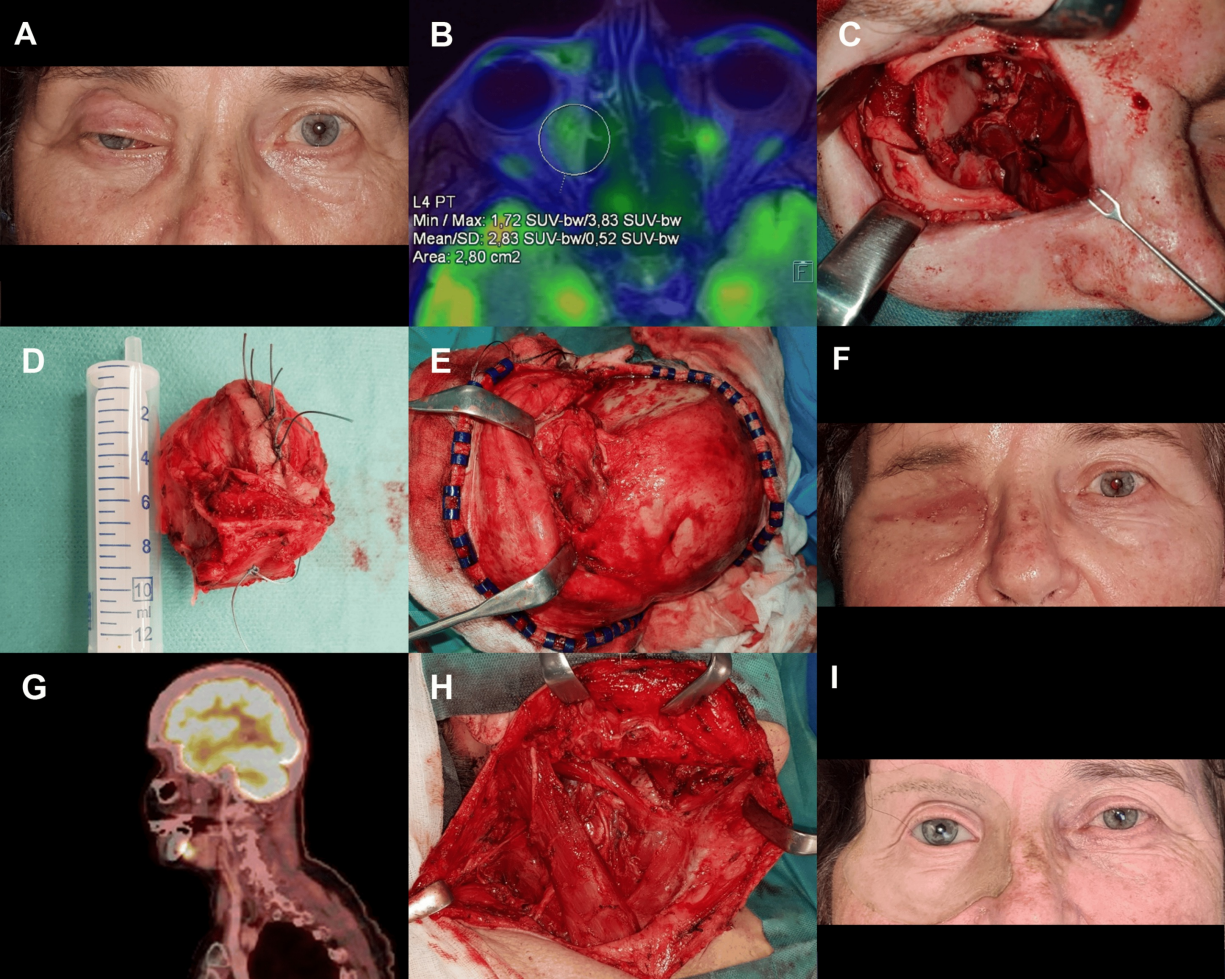
Moderately to poorly differentiated unspecified carcinoma of the right orbit, solid trabecular structure with squamous cell differentiation. (A) Clinical state after non-radical tumour extirpation (R2) at another institution - two months after surgical intervention (B) F-18 fluorodeoxyglucose (18F-FDG) positron emission tomography (PET)/MR axial section, showing two regions of residual tumor tissue in the medial part of the right orbit and the nasolacrimal duct (C) post en bloc orbitectomy of the right orbit (medial, inferior wall and frontal process of maxilla), lateral wall removed for reconstruction (D) surgical specimen (E) reconstruction with temporalis muscle flap (F) post-operative healing, followed by adjuvant radiotherapy of the right orbit (G) 18F-FDG PET/CT sagittal section, after 15 months is showing suspicion of lymph node metastasis in the right submandibular lymph node (Ib) (H) therapeutic neck dissection in the range of ND R (I-V), T0pN2bM0, G3, R0 (metastases in two lymph nodes, area Ib, III) (I) post subsequent adjuvant radiotherapy to the cervical lymph node, orbital epithesis, 67 months with no evidence of disease (NED).

**Figure 2:**
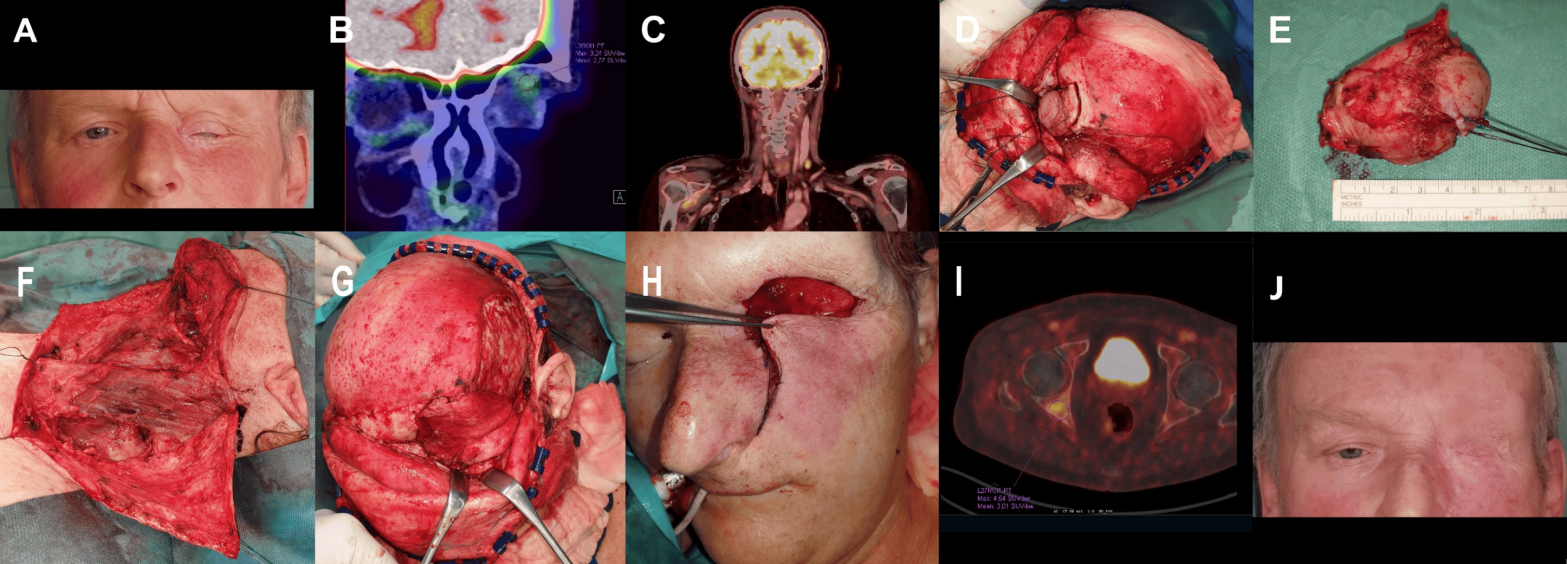
Apocrine adenocarcinoma of the left orbital Moll's gland, intermediate/high grade with lymph angioinvasion and perineural invasion. (A) Clinical state post two non-radical excisions at other institutions and 17 months after proton radiotherapy, left amaurosis (B,C) F-18 fluorodeoxyglucose (18F-FDG) positron emission tomography (PET)/CT coronal sections, core cut biopsy histologically verified tumor showing recurrence under the left orbital roof, suspected lymph node metastasis on the left neck (area IV/Vb) (D) state during en bloc orbitectomy of the left orbit (cranial wall), R1 resection with completion of resection during the operation (E) surgical specimen (F) therapeutic neck dissection in the range of ND L (I-V), histologically proven metastases in two lymph nodes (area IV/Vb) (G, H) reconstruction of the defect with temporalis muscle flap and the skin advancement flap from the left infraorbital region (I) 18F-FDG PET/CT axial section after 22 months, showing metastases in pelvic bones on the right. In addition, metastatic infiltrates intracranially were detected. Died of disease (DOD) 24 months from surgery (J) post-operative healing.

**Figure 3:**
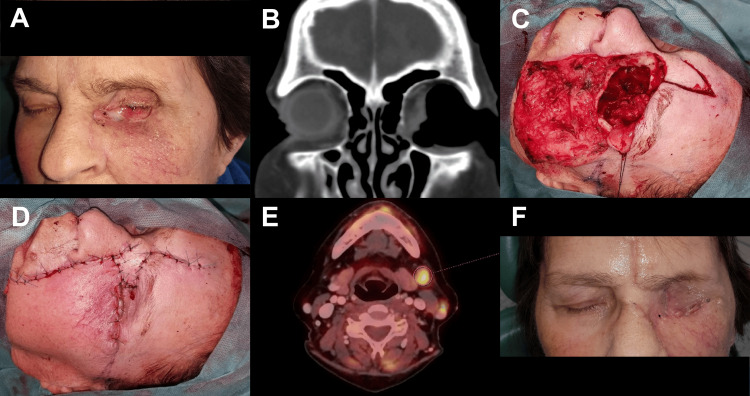
Poorly differentiated sebaceous carcinoma with perineural tumour propagation (A) Clinical state post three non-radical left orbital tumor removals including eyeball enucleation (R1), three months after surgical intervention (B) CT coronal section showing tumor osteolysis of the medial wall of the left orbit (C) post en bloc orbitectomy of the left orbit (medial and inferior wall), R1 resection with completion of resection during the operation (D) reconstruction only with local skin flaps due to the need for a shortened surgical procedure due to polymorbidity (i.a. Coronary artery disease of a transplanted heart) (E) F-18 fluorodeoxyglucose (18F-FDG) positron emission tomography (PET)/CT axial sections showing suspicion of two lymph node metastases in the left neck, not presented at the time of diagnosis, no further treatment options due to ongoing complicated suppurative meningitis, died of other causes (DOC) five months after surgery (F) post-operative healing, followed by adjuvant radiotherapy of the left orbit.

**Figure 4:**
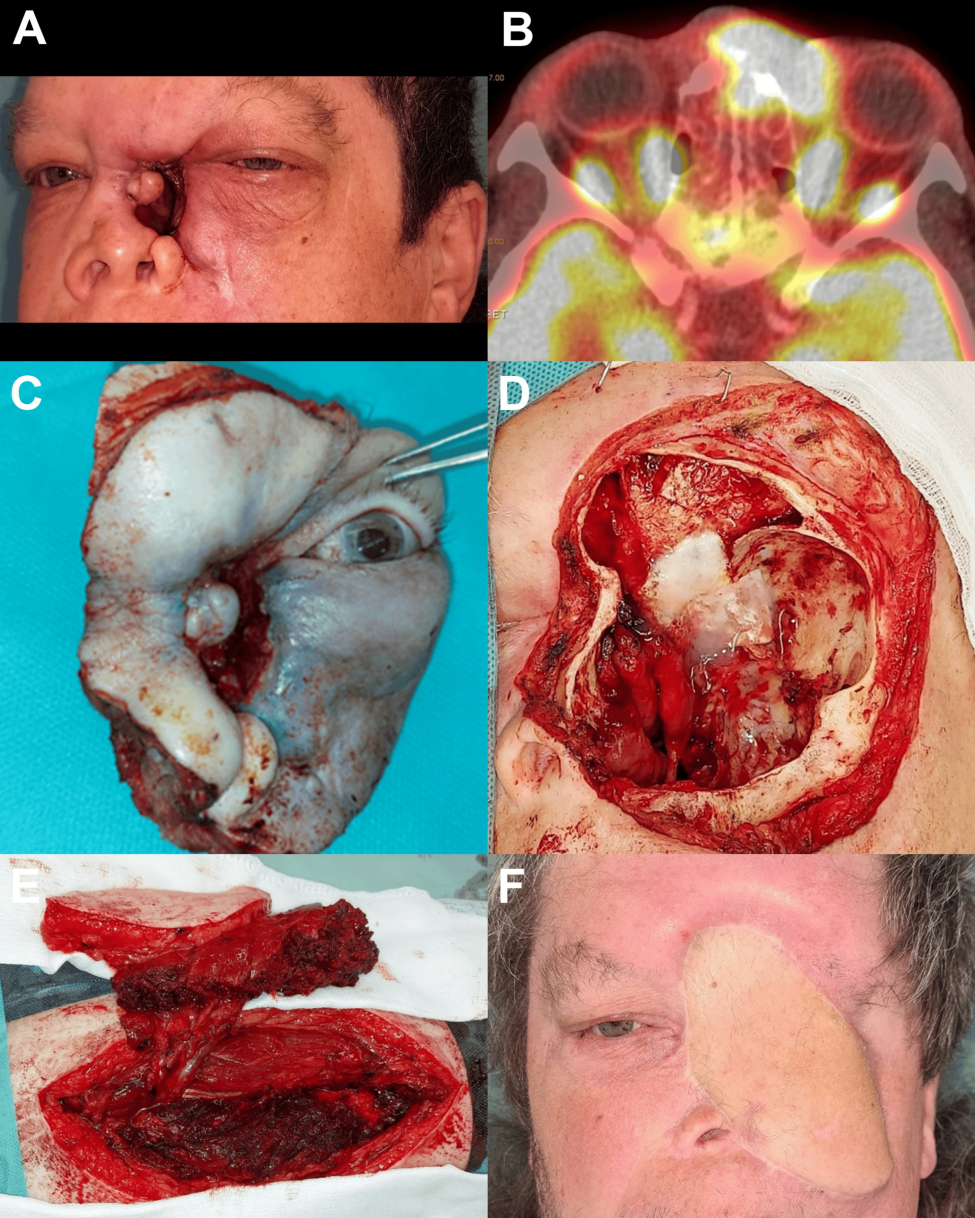
High-grade mucoepidermoid carcinoma with perineural invasion probably arising from the nasal cavity (A) clinical state after non-radical resection at another institution and 14 months after proton radiotherapy (B) F-18 fluorodeoxyglucose (18F-FDG) positron emission tomography (PET)/CT axial section - histologically verified tumour recurrence (C) surgical specimen (D) en bloc orbitectomy of the left orbit (cranial, caudal, and medial wall), R1 resection with completion of resection during the operation. Perioperative cerebrospinal fluid space opening at the anterior margin of the cribriform plate of ethmoid bone was observed - treated with a collagen membrane and tissue glue followed by post-operative five-day external lumbar drainage (E) free anterolateral thigh (ALT) flap ready to be harvested for the reconstruction of the defect (F) clinical state during follow-up. After 24 months, a recurrence in the area of ethmoid and skull base was suspected based on hybrid imaging methods, proton reirradiation therapy was performed, no evidence of disease (NED) 39 months after surgery.

Figures 1-4 show multiple clinical cases of different types of orbital carcinoma with distinct clinical features and prognosis after orbitectomy.

## Discussion

Diagnosing and treating orbital malignancies can be challenging. Prompt diagnosis and treatment are crucial for preserving vision and potentially improving survival rates [13]. Orbital exenteration is categorized into three categories: total, subtotal, and extended. Total exenteration involves the removal of the entire orbital contents along with the eyelid, whereas subtotal or lid-sparing exenteration preserves the eyelids for improved cosmetic outcomes. In cases where disease processes severely affect surrounding structures, extended exenteration (en bloc orbitectomy) is necessary. A multidisciplinary team, typically comprising specialists in head and neck surgery, oncology, pathology, and other relevant fields, is recommended for all cases [14].

En bloc orbitectomy, involving the total orbital contents removal along with bony walls, tends to be a last-resort treatment in clinical practice, employed only after less invasive procedures have failed to manage severe or progressive orbital conditions. In this study, most of the patients (five out of seven) previously underwent multiple less invasive surgeries at other institutions for the same diagnosis. Two patients underwent intended globe-sparing minor surgeries followed by proton beam therapy, which were unsuccessful, leading to en bloc orbitectomy as a salvage surgery. In these patients, radiotherapy led to additional eye complications such as post-radiation keratopathy, secondary glaucoma, and amaurosis, all of which resulted in an aesthetically and functionally unsatisfactory condition, regardless of any recurrence shortly after oncological treatment. These less radical procedures can significantly impact disease control. While aiming to preserve orbital structure and function, they may not fully eradicate malignant tissue, resulting in residual disease and increased risks of recurrence and metastasis. Therefore, opting for orbitectomy as the primary treatment over less radical surgery is crucial in certain cases to ensure the complete removal of malignant tissues and reduce the risk of recurrence.

On the other hand, achieving an R0 resection in an en bloc orbitectomy where the goal is to remove the tumor entirely with no microscopic residual disease presents significant challenges. Therefore, surgical resection should always excise as much tissue as necessary to achieve R0 resection but remove as little healthy tissue as possible to ensure the best possible reconstruction with a good aesthetic outcome; hence, the difficulties in approaching the orbit arise from its confined volume, its irregular four-sided pyramid shape, and its position deeply set within the craniofacial structures [15,16]. Preserving these vital structures while ensuring complete tumor removal requires exceptional surgical planning, precision, and skill. According to our study, R0 resection was achieved in only 50% of the patients, in whom the intention was radical tumor removal. Although in all other cases, an additional radical resection of the tumors was performed during the surgery based on the results of the intraoperative biopsy, these situations cannot be considered anything other than R1 resections. The rate of positive surgical margins for all types of orbital exenteration ranges between 8% and 57.5% in the literature [9]. Achieving clear resection margins is crucial for reducing the risk of local recurrence. However, some studies have shown that orbital exenteration surgery is not associated with lower recurrence rates compared to eye-sparing surgery [9]. The relationship between surgical margins and the prognosis of the disease, especially overall survival, is currently a subject of controversy. In general, the tumor's characteristics (e.g., histological type, size, and tumor spread, including perineural and/or angio-invasion), as well as the patient's biological age and overall health status, play a critical role in determining the life expectancy of patients undergoing orbital surgery. Enhanced multidisciplinary collaboration, including adjuvant therapy consideration and improvements in various areas, including psychological support, surgical recovery, wound care, surgical techniques related to postoperative appearance, and follow-up care, has collectively contributed to a better overall life expectancy [14].

The importance of an overall health assessment before performing an orbitectomy cannot be overstated. In our study, two patients died due to serious complications: one from sepsis 15 days after the operation and another from meningitis following adjuvant radiotherapy. This underscores that a comprehensive preoperative evaluation is essential to ensure the patient is fit for surgery, thereby minimizing potential risks and complications. However, a comprehensive assessment of the individual risks and benefits of surgery is often a challenging task.

The complexities of early identification, accurate staging, and effective treatment monitoring necessitate the utilization of multiple diagnostic imaging techniques. In recent years, advanced hybrid imaging techniques like PET/CT and PET/MRI have played a crucial role in oncologic diagnostics. These modalities are increasingly significant in managing patients with orbital and ocular malignancies [17]. The presence of distant metastases significantly impacts the prognosis for patients with advanced malignant tumors. Proper staging of these metastases is essential for selecting an appropriate treatment plan and predicting patient outcomes. Currently, whole-body tumor staging is clinically performed using tools such as 18F-FDG PET/CT and PET/MRI [18]. They can assist in detecting locoregional primary or nodal recurrences, new distant nodal or organ metastases, and evaluating the response to treatment in the follow-up period [19].

In this study, 18F-FDG PET/CT and 18F-FDG PET/MRI imaging methods were utilized at the time of diagnosis and postoperatively as well to detect locoregional or distant metastasis. These imaging techniques were instrumental in guiding clinical outcomes and monitoring tumor recurrence. Metastasis occurred in four patients: three exhibited locoregional metastasis, and one patient had both locoregional and distant metastasis. As a result, two patients underwent therapeutic en bloc dissection of neck lymph nodes (ND R/L I-V) immediately following orbitectomy as one-stage surgery, based on initial diagnostic imaging at the time of diagnosis. In another patient, this procedure was performed 15 months after orbitectomy after follow-up using 18F-FDG PET/CT scans that suggested potential metastasis.

In a patient with sebaceous carcinoma, an 18F-FDG PET/CT scan revealed suspected lymph node metastases in the left neck five months after surgery, which were not present at the time of diagnosis. However, no further treatment options were indicated due to ongoing complications from suppurative meningitis.

Neck dissection is essential for eliminating and preventing cervical metastasis in many advanced head and neck cancers, which can enhance locoregional control and survival rates. However, the decision to proceed with a neck dissection must also consider the potential morbidity for the patient. In cases of orbital cancer with suspected metastases to regional lymph nodes, typically cervical, based on imaging examinations, therapeutic neck dissection is indicated. Currently, there are no evidence-based guidelines for elective neck dissection in orbital carcinomas, unlike other head and neck tumors such as oral squamous cell carcinoma, where elective neck dissection is recommended if there is a risk of more than 20% occurrence of occult neck metastases [20-22]. The potential for metastasis in orbital carcinomas varies depending on the tumor's characteristics. Certain types of tumors, such as ocular sebaceous carcinoma, are rare but aggressive cancers with a significant risk of locoregional and distant metastasis and can establish regional nodal cervical metastases in 10%-28% of cases [23]. However, in clinical practice, the use of elective neck dissection for this malignancy remains a topic of debate. Although there have been recent improvements, the existing literature primarily consists of case reports of limited size; therefore, it is essential to conduct larger studies with longer follow-up periods to gain a comprehensive understanding of the long-term impacts of different treatment methods [23]. There is ongoing controversy about the necessity of elective neck dissection in orbital cancer due to inconsistent patterns of metastasis and the lack of consensus on predictive indicators for nodal involvement.

Reconstruction after resection of head and neck cancer can be difficult, especially when tumors reach the base of the skull. Over the years, advances in surgical techniques and reconstructive methods have expanded the limits of what is considered feasible for surgical removal, thereby increasing the complexity of reconstruction efforts. Various reconstructive choices come with distinct advantages and drawbacks. Surgical and prosthetic reconstruction aims to reestablish the boundaries between the orbit and adjacent cavities, achieve a satisfactory aesthetic result, and enable easy identification of recurrent disease [24,25]. Skin grafts or local flaps, used to line or fill the orbit, typically accelerate healing, particularly when the orbital roof undergoes resection [26]. Regional flaps are utilized for reconstructing total and extended orbital defects, such as the paramedian frontalis flap, frontalis bilobed flap, glabellar flap, superficial temporalis flap, temporalis muscle flap, and cheek flap [9]. Most of these flaps create bowl-shaped cavities, except for the temporalis muscle flap, which adds fullness to the orbital cavity. Free flaps are used for managing extended orbital exenteration (en bloc orbitectomy) and complex orbital defects, especially when there is a high risk of cerebrospinal fluid (CSF) leakage. Commonly utilized free flaps include the radial forearm flap, anterolateral thigh flap, latissimus dorsi flap, and microvascular scapular flap [9]. In certain situations, it is necessary to perform secondary reconstructions of the orbital region or cosmetic adjustments not only to ensure better condition for orbital epithesis retention. In this study, temporalis muscle flap and local skin flaps were used for most cases for primary reconstruction after the operation, and an ALT-free flap was performed in one patient.

Considering the small sample size and retrospective nature of this study, the study's relatively low number of cases restricts the generalizability of the results, making it challenging to detect significant associations or differences.

Despite these limitations, the study provides preliminary insights into the research topic regarding the benefits of en bloc orbitectomy for achieving long-term disease control, making it a valuable option for patients with malignant orbital carcinomas. Future studies should aim to include larger, more diverse populations and employ prospective designs to enhance the generalizability of the findings.

## Conclusions

The indication for en bloc orbitectomy must be considered carefully and individually when assessing the type and extent of the tumor and the possibility of reconstructing the resulting defect. This procedure aims to thoroughly remove the disease while preserving as much of the patient's natural anatomy and function as possible. Although invasive, orbitectomy can be a vital intervention that offers significant therapeutic benefits, improving outcomes and quality of life for affected patients. A multidisciplinary team makes the decision to perform an orbitectomy, ensuring a comprehensive assessment of the patient's condition and needs.
